# The Current Status of Antioxidants in the Treatment of Vitiligo in China

**DOI:** 10.1155/2022/2994558

**Published:** 2022-02-24

**Authors:** Ying Zhou, Manal Khan, Ling Jiang, Chuhan Fu, Yumeng Dong, Liping Luo, Haoran Guo, Lijuan Gao, Xinxin Lei, Li Zhang, Xing Yu, Li Lei, Jinhua Huang, Jing Chen, Qinghai Zeng

**Affiliations:** Department of Dermatology, Third Xiangya Hospital, Central South University, Changsha, China

## Abstract

Little is known about the use of antioxidants in the clinical treatment of vitiligo. To investigate the specific use of antioxidants in the treatment of vitiligo and the possible reasons behind its use in China, we conducted a prospective questionnaire-based study using an online questionnaire comprising 26 questions in 5 areas. A total of 323 clinical frontline dermatologists participated in this study. Differences among groups were compared using Pearson's chi-square test. Ordinal logistic regression was used to develop knowledge–use multiple regression models. Among the 323 dermatologists, 293 (90.7%) approved the oxidative stress theory of vitiligo, and 182 (56.3%) encouraged the use of antioxidants for treating vitiligo; nonetheless, only 11.8% frequently treated vitiligo with antioxidants. Insufficient knowledge of antioxidants was a significant predictor of lower frequency of antioxidant usage (adjusted odds ratio, 0.401 [95% confidence interval, 0.256-0.629]; *P* < .001). The predictors associated with higher antioxidant efficacy included advanced or rapid progression, moderate or moderate-to-severe vitiligo, age of 0–2 years or 13–18 years, segmental vitiligo, oral and topical combination therapy, and course duration of <1 month. The use of antioxidants for treating vitiligo is highly encouraged; however, the rates of their clinical use are considerably low. Insufficient knowledge of antioxidants is associated with a lower frequency of antioxidant usage. The synergistic curative efficacy of antioxidants could be affected by the stage, type, severity, age of patients with vitiligo, and method of using antioxidants.

## 1. Introduction

Vitiligo is a cutaneous depigmenting disorder resulting from the selective destruction of melanocytes. It affects the mental health of people and decreases their quality of life, thereby hampering their social interactions [1, 2]. In the recent years, studies have investigated the pathogenesis of vitiligo. Although heredity, autoimmunity, psychosis, melanocyte self-destruction, trace element deficiency, and oxidative stress have been recognized as possible determinants of vitiligo, the pathogenesis of this disease is yet to be elucidated [3–7]. Oxidative stress has been shown to be important in the pathogenesis of vitiligo because it damages melanocytes. The following are some potential mechanisms of oxidative stress in vitiligo: [1] oxidative stress injures melanocytes by directly damaging cell membranes, mitochondria, and deoxyribonucleic acid [8]; [2] oxidative stress destroys the structure of melanocytes, thus leading to internal antigen exposure and autoimmune attack [9–11]; [3] oxidative stress contributes to melanocyte apoptosis by inducing the expression of inflammatory factors in keratinocytes and melanocytes [12, 13]; [4] oxidative stress induces the expression of chemokines (CXCL16, CXCL10, etc.) and drives the killing effect of T cells on melanocytes [14, 15]; [5] reactive oxygen species (ROS) reduce tyrosinase activity and inhibit melanin production [16]; and [6] excessive stress can lead to the aging of melanocytes [17]. Therefore, antioxidant strategies may help control the progression of vitiligo.

The consensus or guidelines on vitiligo management in several countries indicated that antioxidant supplements aid in the treatment of vitiligo. A British guideline suggested that *Ginkgo biloba* extract helps prevent the progression of generalized and focal vitiligo [18]. The consensus from China recommends combining antioxidants and phototherapy, particularly during the rapid progression of vitiligo [19]. The consensus from Brazil recommends combining antioxidants and phototherapy in unstable vitiligo [20]. Guidelines from Europe also recommend using antioxidants during ultraviolet (UV) therapy [21]. Several double-blind randomized controlled trials (RCTs) recommend combining narrow-band UVB therapy and antioxidant treatment, such as oral vitamin E [22], oral *Polypodium leucotomos* extract [23], an oral antioxidant pool containing *α*-lipoic acid [24], a topical gel containing catalase and dismutase [25], and topical superoxide dismutase (including copper, zinc, vitamin B12, and calcium pantothenate) [26] for effectively managing vitiligo. Studies have reported that oral *G. biloba* and vitamin E preparations help treat vitiligo even without UV therapy [27, 28].

Although increasing evidence indicates that antioxidants control vitiligo progression and lesion recovery, the frequency of antioxidant use is considerably low in clinical practice. Dermatologists have uncertainties regarding the use and mechanisms of antioxidants, including their efficacy, side effects, and method of use. On the other hand, the patients with vitiligo are worried about the efficacy of antioxidants because of rumors regarding their whitening effect. Thus, we designed this questionnaire to investigate the specific use of antioxidants in the treatment of vitiligo and the possible reasons behind its use in China.

## 2. Methods

### 2.1. Study Design and Participants

We released the questionnaire online to clinical frontline dermatologists in China. Dermatology interns, residents, graduate students, doctoral candidates, and research technicians were not included in this investigation. Data were deidentified and classified to prevent any risk to the participants. We provided ethical informed consent documents to every investigator to ensure that every respondent provided consent. This prospective questionnaire-based study was approved by the Institutional Review Board of the Third Xiangya Hospital of Central South University.

### 2.2. Questionnaires

The Supplemental Questionnaire comprises 26 questions in 5 areas and other information:

(i) (Q1–Q2) Acceptance of the oxidative stress theory of vitiligo pathogenesis and cognition of antioxidants among dermatologists:
  - (Q1) Do you accept the role of oxidative stress in the progression of vitiligo?
  - (Q2) Which of the following do you think are antioxidants?
(ii) (Q3–Q14) Current status of clinical antioxidant application and the factors affecting antioxidant use:
  - (Q3) What is your attitude toward antioxidant use in patients with vitiligo?
  - (Q4–Q5) Have you ever used antioxidants in clinically treating vitiligo? Evaluate the proportion
  - (Q6–Q8) Does the stage, type, or area of the skin lesions of vitiligo in a patient affect your choice on the use of antioxidants?
  - (Q9–Q11) In which stages, types, or areas of the skin lesions of vitiligo do you consider using antioxidants?
  - (Q12) In which age group of patients do you consider using antioxidants?
  - (Q13–Q14) How and how long do you use antioxidants?
(iii) (Q15–Q20) Evaluation of the efficacy and side effects of antioxidants:
  - (Q15) In your clinical experience, do antioxidants have synergistic therapeutic effects in the overall assessment?
  - (Q16–Q17) Do patients report side effects when using antioxidants? Evaluate the proportion
  - (Q18–Q19) Have patients reported whitening or enlargement of leukoplakia during the use of antioxidants? Evaluate the proportion
  - (Q20) Which of the following side effects did the patients report?
(iv) (Q21) Reasons for refusing antioxidant use:
  - (Q21) Which of the following are your possible reasons for not using antioxidants?
(v) (Q22–Q26) The need for guidelines or consensus for the antioxidant management of vitiligo:
  - (Q22) Do you wish expert consensus or guidelines for the use of antioxidants?
  - (Q23) Would you use antioxidants if RCTs or medical evidence supporting the effectiveness of antioxidants for treating vitiligo were available?
  - (Q24) Would you use antioxidants if consensus or guidelines recommend antioxidants for treating vitiligo?
  - (Q25–Q26) Are there any patients with vitiligo or their family members who consulted about the edibility of foods rich in vitamin C in your clinical work? What is your answer?

### 2.3. Questionnaire Data Extraction and Data Definition

The frequency of antioxidant use was defined as “never”=1, “occasionally”=2, or “frequently or always”=3. Antioxidant synergy was defined as “markedly effective”=1, “effective”=2, or “uncertain”=3. Hospital grades were defined as “tertiary A hospital”=1, “tertiary hospital”=2, “secondary hospital”=3, or “first-level hospital and below”= 4. In the antioxidant cognition test item, we established a multiple-choice question with seven options: vitamin C, vitamin E, reduced glutathione, tea polyphenols, resveratrol, *G. biloba* extract, and *Rhodiola* extract. We then let respondents determine which substance is an antioxidant. All of these options are correct answers; i.e., all options are antioxidants. We gave a score of 1 for correct answers and 0 for incorrect answers. Overall scores of 1–4 and overall scores of 5–7 reflected “insufficient” and “sufficient” knowledge of antioxidants, respectively.

### 2.4. Statistical Methods

Measurement data were analyzed using the *t*-test, and differences among groups were compared using Pearson's chi-square test. Ordinal logistic regression was used to develop knowledge–use multiple regression models. The retention of covariates in the multivariate models was based on the consideration of confounding and effect modifications. Sex, current age (continuous), and hospital level were evaluated individually and in combination. Adjusted odds ratios (aORs) were calculated by including them in the full model. All data processing and statistical analyses were performed using commercially available software (SPSS, version 25, IBM Corp, Armonk, NY, USA). All *P* values were two-tailed, and *P* = .05 was the threshold for statistical significance. The multiple dependent tests performed in this study increased the risk of falsely rejecting the null hypothesis. Therefore, *P* values close to.05 should be interpreted with caution.

## 3. Results

### 3.1. Characteristics of the Respondents

We investigated the characteristics of the respondents, including sex, age, education background, doctor title, and hospital level. Among the 323 responding dermatologists from 145 medical institutions in China, 35.3% and 64.7% were female and male, respectively. The age range of the respondents was 26–60 years (97.2%). The respondents mainly had master's degrees (39.3%), and a majority (81.5%) practiced in tertiary care hospitals (Supplemental Figure 1).

### 3.2. Acceptance of the Oxidative Stress Theory of Vitiligo and Knowledge of Antioxidants among Dermatologists

**Figure 1:**
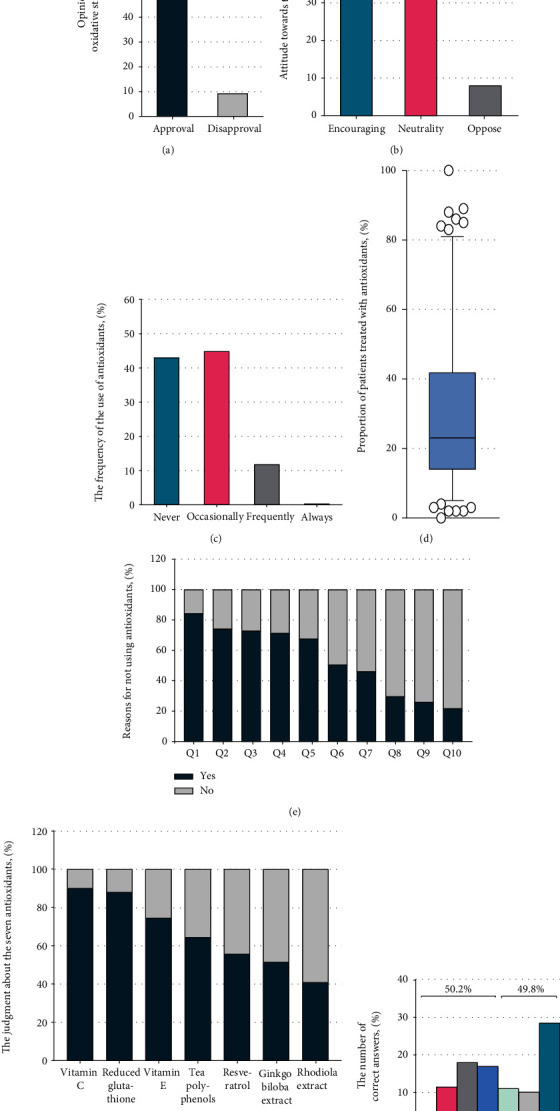
Acceptance of the oxidative stress theory of vitiligo and knowledge of antioxidants among dermatologists. (a) Opinion of dermatologists on the oxidative stress theory of vitiligo. (b) Attitude toward antioxidant use among dermatologists. (c) Frequency of antioxidant use among dermatologists for treating vitiligo. (d) Proportion of dermatologists who used antioxidants clinically and proportion of patients treated with antioxidants. (e) Reasons for not using antioxidants. q1: Is it because of the uncertain efficacy of antioxidants? (yes: 117 dermatologists [84.2%]; no: 22 dermatologists [15.8%]). q2: Is it because of the controversial evidence of antioxidants mentioned in dermatology-related textbooks or literature? (yes: 103 dermatologists [74.1%]; no: 36 dermatologists [25.9%]). q3: Is it because of the lack of knowledge about antioxidants? (yes: 101 dermatologists [72.7%]; no: 38 dermatologists [27.3%]). q4: Is it because of whitening effects or other scientific theories of antioxidants? (yes: 99 dermatologists [71.2%]; no: 40 dermatologists [28.8%]). q5: Is it because of worrying about the misunderstanding of patients, which may lead to medical disputes? (yes: 94 dermatologists [67.6%]; no: 45 dermatologists [32.4%]). q6: Is it because of unsuitable antioxidants in the hospital? (yes: 70 dermatologists [50.4%]; no: 69 dermatologists [49.6%]). q7: Is it because of worrying about the side effect of antioxidants? (yes: 64 dermatologists [46.0%]; no: 75 dermatologists [54.0%]). q8: Is it because of the indeterminate methods for using antioxidants? (yes: 41 dermatologists [29.5%]; no: 98 dermatologists [70.5%]). q9: Other reasons (yes: 36 dermatologists [25.9%]; no: 103 dermatologists [74.1%]). q10: Is it because of the price of antioxidants? (yes: 30 dermatologists [21.6%]; no: 109 dermatologists [78.4%]). (f) Answers of the dermatologists about the seven antioxidants (vitamin C, reduced glutathione, vitamin E, tea polyphenols, resveratrol, *Ginkgo biloba* extract, and *Rhodiola* extract). (g) Number of correct answers for antioxidant-related questions among dermatologists.

Among the 323 respondents, 293 respondents (90.7%) approved the oxidative stress theory of vitiligo (Figure 1(a)), whereas 56.3% encouraged treating vitiligo with antioxidants (Figure 1(b)). Surprisingly, in clinical practice, only 39 respondents (12.1%) treated vitiligo with antioxidants frequently or always, and 139 (43%) never used antioxidants for treating vitiligo (Figure 1(c)). A total of 184 respondents (57%) treated vitiligo with antioxidants, and the proportion of patients treated with antioxidants was 30.82% (Figure 1(d)).

We investigated the underlying causes among dermatologists who refused to use antioxidants and found that the leading reason was the uncertain efficacy of antioxidants (q1, 84.2%), followed by controversial evidence of antioxidant efficacy in dermatology-related textbooks or literature (q2, 74.1%) and insufficient knowledge of antioxidants (q3, 72.7%) (Figure 1(e)). Furthermore, we described possible clinical situations that affect antioxidants use (Supplemental Figure 2), while further studies are needed to confirm the association between the clinical situations and antioxidants use. Then, we tested whether the participating dermatologists can accurately determine that the seven real antioxidants are antioxidants. The accuracy in determining vitamin C, vitamin E, reduced glutathione, and tea polyphenols as antioxidants was more than 60% (90.1%, 74.6%, 87.9%, and 64.4%, respectively). The accuracy in determining resveratrol, *G. biloba* extract, and *Rhodiola* extract as antioxidants was lower than 60% (55.7%, 51.4%, and 40.9%, respectively) (Figure 1(f)). Surprisingly, the rate of insufficiency of knowledge of antioxidants reached 50.2%, and the rate of sufficient knowledge reached 49.8% (Figure 1(g)).

### 3.3. Knowledge–Use Multiple Regression Models

To analyze the association between the demographic variables, the frequency of antioxidants use, and the synergistic curative efficacy of antioxidants, we performed chi-square tests between five demographic variables (sex, age, education, hospital level, and title of the dermatologist) and two dependent variables (frequency and efficacy of antioxidants). The results indicated that two demographic variables (sex and hospital level) were significantly associated with the dependent variables described above (*P* < .05) (Supplemental Tables 1–2). Therefore, all of these were considered potential confounding variables and were included in the multivariate analyses.

We firstly explored the association between the knowledge of antioxidants and the frequency of antioxidants use in bivariate models; the frequency of antioxidant use among dermatologists was significantly associated with knowledge of antioxidants (*P* ≤ .001) (Supplemental Table 3). We then explored knowledge–use regression models on the basis of the results. Three different multivariate logistic regression models were created: [1] use evaluation models of hospital level, sex, and age; [2] knowledge evaluation model of hospital level, sex, and age; and [3] knowledge–use regression models of hospital level, sex, age, and the knowledge of antioxidants. In model 1, male sex was a predictor of higher frequency of antioxidant use (aOR, 1.844 [95% confidence interval [CI], 1.165-2.917]; *P* = .009). Conversely, in model 2, male sex was a predictor of lesser knowledge of antioxidants (aOR, 0.474 [95% CI, 0.290-0.776]; *P* = .003), and age was a predictor of higher knowledge of antioxidants. In model 3, male sex was a predictor of higher frequency of antioxidant use (aOR, 2.199 [95% CI, 1.369-3.532]; *P* ≤ .001), and insufficient knowledge of antioxidants was a predictor of lower frequency of antioxidant use (aOR, 0.401[95% CI, 0.256-0.629]; *P* < .001).

Overall, in the multivariable linear regression analysis, age was a significant predictor of higher knowledge of antioxidants. Male sex was a significant predictor of lower knowledge of antioxidants; nevertheless, males had the opposite effect in terms of the frequency of antioxidant use. The effect of the male variable was still significant after knowledge of antioxidants was added as a variable to model 3. Finally, insufficient knowledge of antioxidants was a significant predictor of lower frequency of antioxidant use (Table 1). This means that a higher knowledge of antioxidants is correlated with a higher frequency of antioxidant use.

### 3.4. Evaluated Antioxidant Synergistic Curative Efficacy and the Possible Risk Factors

**Figure 2:**
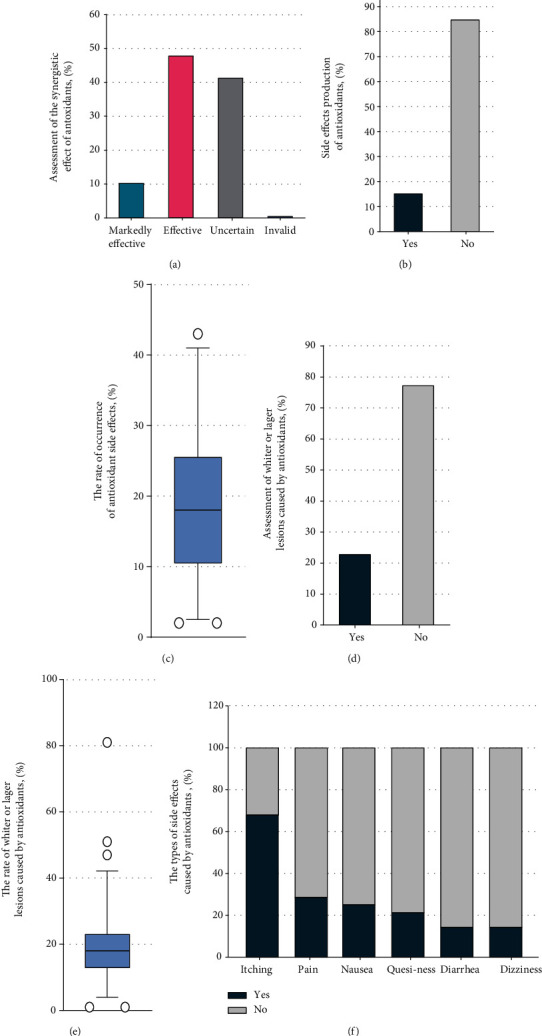
Assessment of the synergistic effect of antioxidants and the possible side effects. (a) Assessment of the synergistic effect of antioxidants. (b) Assessment of the side effects of antioxidants. (c) Assessment of the rate of occurrence of antioxidant side effects. (d) Assessment of whiter or lager lesions caused by antioxidants. (e) Assessment of the rate of occurrence of whiter or larger lesions caused by antioxidants. (f) Assessment of the types of side effects caused by antioxidants.

We evaluated the antioxidant synergistic curative efficacy among those who used antioxidants (*N* = 184). Most respondents (47.8%) assessed the antioxidant synergistic curative efficacy as effective, 76 (41.3%) assessed it as uncertain, and only 1 (0.5%) assessed it as invalid (Figure 2(a)). Most of the respondents (84.8%) assessed the side effects of antioxidants as no side effects (Figure 2(b)). A total of 28 respondents (15.2%) assessed that antioxidants had side effects, and the proportion of patients who experienced side effects was 18.96% (Figure 2(c)). A total of 142 respondents (77.2%) assessed that no whiter or larger lesions were observed after using antioxidants (Figure 2(d)). A total of 42 respondents (22.8%) stated that they had encountered patients who described to them about observing a whiter or larger lesion after using antioxidants. And the 42 respondents reported that the proportion of patients describing whiter or larger lesions after using antioxidants was 20.52% (Figure 2(e)). A total of 28 respondents (15.2%) assessed that the main side effects of antioxidants were pruritus, pain, and nausea (Figure 2(f)).

To further determine the factors that influence the synergistic efficacy of antioxidants, a chi-square test was performed for screening differential variables. Variables associated with the synergistic efficacy of antioxidants were advanced or rapid progression, moderate or moderate to severe vitiligo, age of 0–2 years or 13–18 years, segmental vitiligo, oral and topical combination therapy, and a course duration of <1 month (*P* ≤ 0.037). All of these were therefore considered potential predictors and were included in the multivariate analyses (Supplemental Table 4). In bivariate models, the association between the antioxidant synergistic curative efficacy and the following clinical situations had significantly higher crude odds ratio (OR) (advanced progression of vitiligo: crude OR, 2.805 [95% CI, 1.558-5.053], *P* ≤ .001; rapid progression of vitiligo: crude OR, 2.196 [95% CI, 1.238-3.895], *P* = .007; segmental vitiligo: crude OR, 2.381 [95% CI, 1.332-4.258], *P* = .003; moderate vitiligo: crude OR, 2.630 [95% CI, 1.428-4.845], *P* = .002; moderate to severe vitiligo: crude OR, 2.489 [95% CI, 1.405-4.408], *P* = .002; age of 0-2 years: crude OR, 3.405 [95% CI, 1.237-9.373], *P* = .018; age of 13-18 years: crude OR, 2.108 [95% CI, 1.182-3.758], *P* = .011; and oral and topical combination therapy: crude OR, 2.668 [95% CI, 1.485-4.794], *P* = .001) (Table 2). Antioxidant synergistic curative efficacy had a stronger association with a course duration of <1 month (aOR, 18.648 [95% CI, 3.629-95.822]; *P* ≤ .001) than with a course duration of >1 month. These associations remained significant, including the hospital level, sex, and current age (*P* < .016); that is, the situations described above and a duration of <1 month of the course were significant predictors of higher antioxidant efficacy.

### 3.5. Attitude toward Guidelines, Medical Evidence, and Edibility of Antioxidant-Related Food

**Figure 3:**
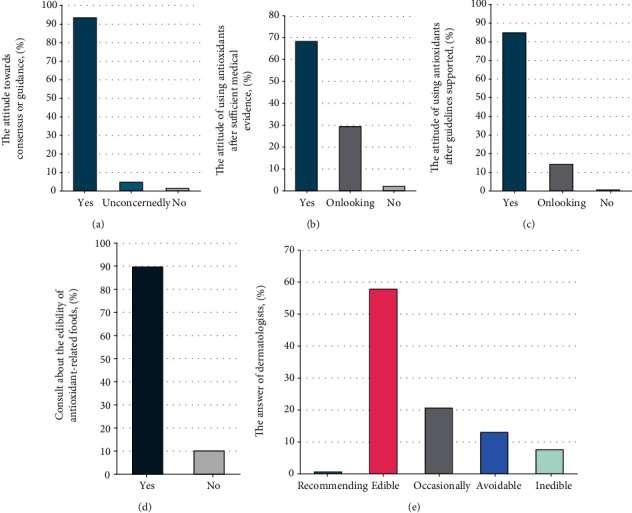
Attitude toward guidelines, medical evidence, and edibility of antioxidant-related foods. (a) Attitude of dermatologists toward consensus or guidance. (b) Attitude of dermatologists toward antioxidant use after the provision of sufficient medical evidence. (c) Attitude of dermatologists toward antioxidant use after the provision of guidelines. (d) Patients or their family members who consulted about the edibility of antioxidant-related foods. (e) The answer of dermatologists regarding the edibility of antioxidant-related foods.

The data showed that over 93.5% of the respondents hoped for a consensus or guidelines on the use of antioxidants in vitiligo (Figure 3(a)). Among the respondents, 68.3% and 84.9% showed a willingness to use antioxidants after sufficient medical evidence, and guidelines are provided for the use of antioxidants, respectively (Figures 3(b) and 3(c)). One of the interesting findings was that dermatologists and patients or their family members were concerned with the use of antioxidants for vitiligo, and 290 respondents (89.8%) indicated that patients or their family members consulted them about the edibility of antioxidant-related food (Figure 3(d)). A total of 2 (0.7%), 168 (57.9%), 60 (20.7%), 38 (13.1%), and 22 (7.6%) respondents answered that antioxidant-related food is recommending edible, edible, occasionally edible, avoidable, and inedible, respectively (Figure 3(e)). Overall, consensus had a higher approval for guiding the use of antioxidants in vitiligo than medical evidence from dermatologists.

## 4. Discussion

Clinically, dermatologists seemed to face a “usage paradox”; that is, antioxidant use has stagnated despite support toward the oxidative stress theory and the use of antioxidants for treating vitiligo. Among the 323 dermatologists, 293 (90.7%) approved the oxidative stress theory of vitiligo, and 182 (56.3%) encouraged the use of antioxidants for treating vitiligo; nonetheless, only 11.8% of them frequently treated vitiligo with antioxidants. We investigated the possible reasons behind this paradox, and the data showed that the uncertain efficacy of antioxidants (84.2%) was the leading cause, followed by controversial evidence of antioxidant efficacy in dermatology-related textbooks or literature (74.1%) and insufficient knowledge of antioxidants (72.7%). Encouragingly, 118 respondents (84.9%) showed a willingness to use antioxidants with guidelines, which can be an effective solution. A study showed that the treatment of vitiligo by dermatologists is complex and generally unsatisfactory [29]. When prescribing antioxidants, dermatologists should first consider the best interest of the patient rather than sociocultural factors; however, in addition to the therapeutic effects of drugs, dermatologists usually consider all individual outcomes and psychological and sociocultural factors [30–33].

A study showed that knowledge of drugs affected decisions concerning clinical treatment; however, the relevance between knowledge of drugs and use of drugs was not mentioned [34]. In the current study, insufficient knowledge of antioxidants was a significant predictor of a lower frequency of antioxidant use (aOR, 0.401 [95% CI, 0.256-0.629]; *P* < .001). Interestingly, knowledge did not differ significantly between junior and senior dermatologists, thus indicating that education should target both groups; this finding is consistent with data from a similar study [35]. Most educational sessions are designed for residents; however, senior dermatologists often decide whether hospitalized patients require antioxidants and which antioxidants should be used [36].

Recent studies indicated that guidelines affect treatment decisions [37, 38]. Our data indicated that the controversial evidence of antioxidant efficacy in dermatology-related textbooks or literature (74.1%) affects antioxidant usage, 93.5% of dermatologists hoped for consensus or guidelines for the use of antioxidants in vitiligo, and antioxidant usage would increase with guidelines (84.9%) or sufficient medical evidence (68.3%). The guidelines are expected to eliminate bias caused by age and sex regarding the knowledge of antioxidants and enhanced usage of antioxidants. In addition, factors that increase antioxidant usage include sharing of best practices among institutions, use of resources to answer knowledge-related questions, and use of guidelines and smartphone applications as frequent sources for learning about antioxidants.

Although the updated guidelines recommend antioxidant supplements for treating vitiligo, its specific usage is still unclear [19]. The predictors associated with higher antioxidant efficacy included advanced or rapid progression, moderate or moderate-to-severe vitiligo, age of 0–2 years or 13–18 years, segmental vitiligo, oral and topical combination therapy, and course duration of <1 month. A study showed that patients with active vitiligo had a higher level of ROS than patients with stable vitiligo [39]. Therefore, in cases of advanced progression, rapid progression, moderate vitiligo, and moderate-to-severe vitiligo, patients would be more susceptible to antioxidative stress and would benefit more from antioxidants. An RCT on childhood vitiligo showed that topical antioxidant pseudocatalase PC-KUS successfully treated vitiligo [40]. Topical antioxidants alone rarely succeed in treating vitiligo, fail to show reproducible results [41], and cannot fully restore the balance of the intracellular redox status [24]. Therefore, oral and topical combination therapy is a better option for treating vitiligo. Our data showed that a course of <1 month was a better option for treating vitiligo. However, further research is required to confirm these results.

Although studies have suggested that antioxidant supplementation plays a therapeutic role in various diseases without side effects, such as adverse events in adult surgical patients [42], inflammatory lung diseases [43], and neurotoxicity induced by platinum-based chemotherapeutics [44], the side effects of antioxidants are still controversial. Some studies reported that zinc as an antioxidant can cause additional mental stress [45], and some strong oxidant compounds containing phenol groups were reported to cause chemical vitiligo [46, 47]. Our data showed that the major side effects of antioxidants were pruritus, pain, and nausea. Indeed, the selection of appropriate antioxidants, knowledge of definite side effects, and reduction of side effects can help improve the use of antioxidants.

## 5. Conclusion

We identified the possible reasons for the paradox of antioxidants being highly recommended in vitiligo treatment but having low rates of use in clinical practice. Our results could help in designing specific education programs and updating guidelines aimed at improving the use of antioxidants.

## Figures and Tables

**Table 1:** Analysis of the association between knowledge of antioxidants, confounding variables, and antioxidant usage among dermatologists.

Variable	Model 1^b^		Model 2^a^		Model 3^b^	
	aORs (95% CI)	*P* value	aORs (95% CI)	*P* value	aORs (95% CI)	*P* value
Knowledge of antioxidants						
Insufficient	NI	NA	NI	NA	0.401 (0.256-0.629)	<.001
Sufficient	NI	NA	NI	NA	1 [reference]	NA
Hospital level						
Tertiary A hospital	1.105 (0.452-2.706)	.826	0.785 (0.303-2.037)	.619	1.138 (0.459-2.820)	.781
Tertiary hospital	2.541 (0.934-6.911)	.068	1.255 (0.430-3.658)	.678	2.482 (0.901-6.833)	.079
Secondary hospital	1.055 (0.372-2.988)	.920	0.641 (0.211-1.952)	.434	1.111 (0.387-3.190)	.845
First-level hospital and below	1 [reference]	NA	1 [reference]	NA	1 [reference]	NA
Sex						
Male	1.844 (1.165-2.917)	.009	0.474 (0.290-0.776)	.003	2.199 (1.369-3.532)	≤.001
Female	1 [reference]	NA	1 [reference]	NA	1 [reference]	NA
Age	0.954 (0.764-1.190)	.676	1.376 (1.082-1.752)	.009	0.893 (0.712-1.121)	.330

Abbreviations: aOR, adjusted odds ratio; NA, not applicable; NI, not included. ^a^Binary logistic regression model was constructed with knowledge of antioxidants (insufficient and sufficient) as the binary dependent (outcome) variable. Model 2 included the potential confounding variables. ^b^Ordinal logistic regression models were constructed with the frequency of the use of antioxidants (never, occasionally, frequently, or always) as the ordinal dependent (outcome) variable in models 1 and 3. Model 1 included the potential confounding variables. Model 3 included random effects for the knowledge of antioxidants. In models 1, 2, and 3, the hospital level, sex, and current age were included as covariates. In model 3, knowledge of antioxidants was also included as a covariate. There were no significant interactions between knowledge of antioxidants or use of antioxidants and other covariates in the multivariate models.

**Table 2:** Possible significant predictors of the synergistic curative efficacy of antioxidants.

Variable	Treat with antioxidants (*N* = 184)^a^				
	Freq, NO. (%)	Crude OR (95% CI)	*P* value	aORs (95% CI)	*P* value
Advanced progression					
Nonuse	75 (40.8)	2.805 (1.558-5.053)	≤.001	2.768 (1.509-5.076)	≤.001
Use	109 (59.2)	1 [reference]	NA	1 [reference]	NA
Rapid progression					
Nonuse	104 (56.5)	2.196 (1.238-3.895)	.007	2.570 (1.395-4.736)	.002
Use	80 (43.5)	1 [reference]	NA	1 [reference]	NA
Segmental vitiligo					
Nonuse	74 (40.2)	2.381 (1.332-4.258)	.003	2.587 (1.427-4.689)	.002
Use	110 (59.8)	1 [reference]	NA	1 [reference]	NA
Moderate vitiligo (area 1%-5%)					
Nonuse	63 (34.2)	2.630 (1.428-4.845)	.002	2.384 (1.273-4.467)	.007
Use	121 (65.8)	1 [reference]	NA	1 [reference]	NA
Moderate to severe vitiligo (area 6%-50%)					
Nonuse	92 (50.0)	2.489 (1.405-4.408)	.002	2.944 (1.610-5.382)	<.001
Use	92 (50.0)	1 [reference]	NA	1 [reference]	NA
0–2 years old patients					
Nonuse	168 (91.3)	3.405 (1.237-9.373)	.018	3.879 (1.382-10.888)	.010
Use	16 (8.7)	1 [reference]	NA	1 [reference]	NA
13–18 years old patients					
Nonuse	113 (61.4)	2.108 (1.182-3.758)	.011	2.046 (1.141-3.668)	.016
Use	71 (38.6)	1 [reference]	NA	1 [reference]	NA
Oral and topical combination therapy					
Nonuse	109 (59.2)	2.668 (1.485-4.794)	.001	2.768 (1.514-5.061)	.001
Use	75 (40.8)	1 [reference]	NA	1 [reference]	NA
The course duration of antioxidants(months)					
< 1	16 (8.7)	16.341 (3.357-79.543)	.001	18.648 (3.629-95.822)	<.001
1–3	122 (66.3)	2.621 (0.953-7.211)	.062	2.459 (0.887-6.817)	.084
4–6	30 (16.3)	1.822 (0.564-5.883)	.316	1.914 (0.588-6.223)	.281
> 6	16 (8.7)	1 [reference]	NA	1 [reference]	NA

Abbreviations: aOR, adjusted odds ratio; NA, not applicable; Freq, frequency. ^a^Ordinal logistic regression models were constructed with the synergistic effect of antioxidants (markedly effective, effective, or uncertain) as the ordinal dependent (outcome) variable. The independent (explanatory) variable was the combined classification of vitiligo, the area affected by vitiligo, the age of the patients, the pattern of antioxidant use, and the duration of the course (categorical). The crude OR (95% confidence interval) for the synergistic curative efficacy of the antioxidants was determined. The aORs were determined from multivariate models by including the hospital level, sex, and current age. There were no significant interactions between the classification of vitiligo, the area affected by vitiligo, the age of the patients, the pattern of antioxidant use, the duration of the course, and the other covariates in the multivariate models.

## Data Availability

The data used to support the findings of this study are available from the corresponding author upon request.

## Supplementary Materials

Supplementary 1 Supplementary questionnaire.

Supplementary 2 Supplemental Figure 1: the demographic distribution of the respondents.

Supplementary 3 Supplemental Table 1: analysis of the association between the demographic variables and the frequency of the use of antioxidants.

Supplementary 4 Supplemental Table 2: analysis of the association between the demographic variables and the synergistic curative efficacy of antioxidants.

Supplementary 5 Supplemental Table 3: analysis of the association between the knowledge of antioxidants and the frequency of the use of antioxidants.

Supplementary 6 Supplemental Table 4: analysis of the association between the clinical situations and the synergistic curative efficacy of antioxidants.

Supplementary 7 Supplemental Figure 2: possible clinical situations affect the choice for the use of antioxidants.
