# Supplementary material for: The Current Status of Antioxidants in the Treatment of Vitiligo in China
Source: Oxid Med Cell Longev. 2022 Feb 24;2022:2994558. doi: 10.1155/2022/2994558 (PMC8896159; doi:10.1155/2022/2994558)
Supplement: Supplementary 1 — Supplementary questionnaire. [file 2994558.f1.docx]

Supplemental Questionnaire

A survey of state quo of use of antioxidants in the treatment of vitiligo

1. Do you accept the role of oxidative stress in the progression of vitiligo?

a. approval

b. disapproval

2. Which of the following do you think are antioxidants?

a. vitamin C

b. vitamin E

c. reduced glutathione

d. tea polyphenols

e. resveratrol

f. Ginkgo biloba extract

g. Rhodiola extract

3. What is your attitude toward antioxidant use in patients with vitiligo?

a. encouraging

b. neutrality

c. oppose

4. Have you ever used antioxidants in clinically treating vitiligo?

a. never

b. occasionally

c. frequently

d. always

5. What percentage of patients with vitiligo do you treat with antioxidants? (Enter the number from 0 to 100).

6. Does the stage of vitiligo in a patient (stable period or advanced period) affect your choice for the use of antioxidants?

a. yes

b. no

7. Does the type of patient (segmental, non-segmental, or undetermined type) affect your choice for the use of antioxidants?

a. yes

b. no

8. Does the area of the skin lesions in a patient (light, moderate, moderate to severe, or severe) affect your choice for the use of antioxidants?

a. yes

b. no

9. In which stages of vitiligo do you consider using antioxidants?

a. stable progression

b. advanced progression

c. rapid progression

10. In which type of patients do you consider using antioxidants?

a. segmental vitiligo

b. non-segmental vitiligo

c. undetermined vitiligo

11. In which areas of the skin lesions do you consider using antioxidants?

a. mild vitiligo (area < 1%)

b. moderate vitiligo (area 1%-5%)

c. moderate to severe vitiligo (area 6%-50%)

d. severe vitiligo (area > 50%)

12. In which age group of patients do you consider using antioxidants? (Q12);

a. 0–2 years

b. 3–12 years

c. 13–18 years

d. >18 years

13. How do you use antioxidants? (Q13);

a. topical therapy

b. oral therapy

c. oral and topical combination therapy

14. For how long do you usually use antioxidants? (Q14);

a. < 1 month

b. 1–3 months

c. 4–6 months

d. >6 months

15. In your clinical experience, do antioxidants have synergistic therapeutic effects in the overall assessment?

a. markedly effective

b. effective

c. uncertain

d. invalid

16. Do patients report side effects when using antioxidants?

a. yes

b. no

17. Evaluate the proportion of patients with adverse effects (Enter the number from 0 to 100).

18. Have patients reported whitening or enlargement of leukoplakia during the use of antioxidants?

a. yes

b. no

19. The percentage of patients with whitening or enlargement of leukoplakia during antioxidant use (Enter the number from 0 to 100).

20. Which of the following side effects did the patients report?

a. itching

b. pain

c. nausea

d. queasiness

e. diarrhea

f. dizziness

21. Which of the following are your possible reasons for not using antioxidants?

q1. Is it because of the uncertain efficacy of antioxidants?

a. yes

b. no

q2. Is it because of the controversial evidence of antioxidants mentioned in dermatology-related textbooks or literature?

a. yes

b. no

q3. Is it because of the lack of knowledge about antioxidants?

a. yes

b. no

q4. Is it because of whitening effects or other scientific theories of antioxidants?

a. yes

b. no

q5. Is it because of worrying about the misunderstanding of patients, which may lead to medical disputes?

a. yes

b. no

q6. Is it because of unsuitable antioxidants in the hospital?

a. yes

b. no

q7. Is it because of worrying about the side effect of antioxidants?

a. yes

b. no

q8. Is it because of the indeterminate methods for using antioxidants?

a. yes

b. no

q9. other reasons.

a. yes

b. no

q10. Is it because of the price of antioxidants?

a. yes

b. no

22. Do you wish expert consensus or guidelines for the use of antioxidants?

a. yes

b. unconcernedly

c. no

23. Would you use antioxidants if RCTs or medical evidence supporting the effectiveness of antioxidants for treating vitiligo were available?

a. yes

b. onlooking

c. no

24. Would you use antioxidants if consensus or guidelines recommend antioxidants for treating vitiligo?

a. yes

b. onlooking

c. no

25. Are there any patients with vitiligo or their family members who consulted about the edibility of foods rich in vitamin C in your clinical work?

a. yes

b. no

26. What is your answer when patients with vitiligo or their family members ask you about the edibility of foods rich in vitamin C?

a. recommending

b. edible

c. occasionally edible

d. avoidable

e. inedible


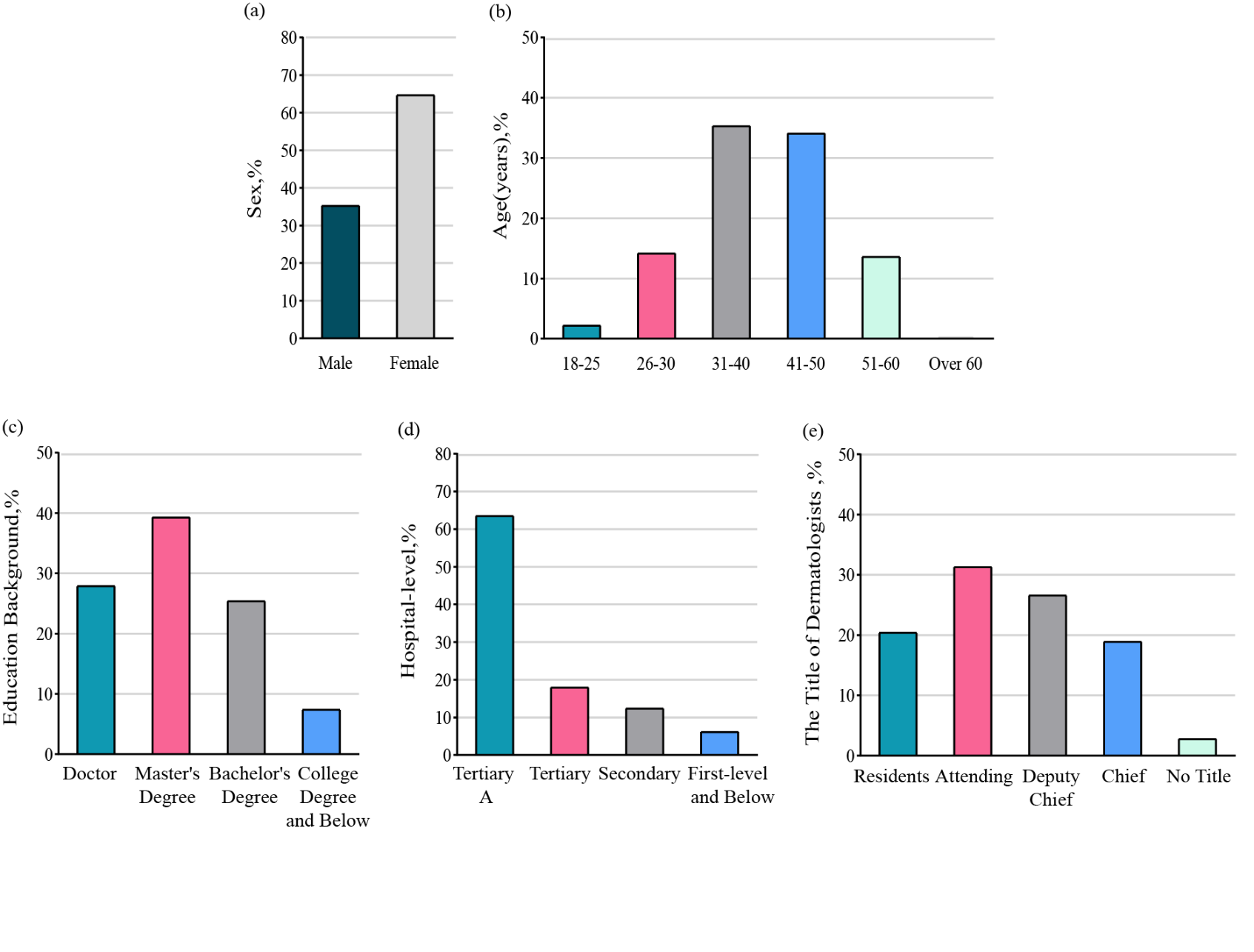


Supplemental Figure 1. The Demographic Distribution of The Respondents.

(a), The distribution of sex of the respondents; (b), The distribution of the age of the respondents; (c), The distribution of educational background of the respondents; (d), The distribution of hospital level of the respondents; (e), The distribution of the title of the respondents.

Supplemental Table 1. Analysis of the Association Between the Demographic Variables and the Frequency of the Use of Antioxidants.

| Characteristic ^b^ | Never use antioxidants | | Use antioxidants occasionally | Always use antioxidants | $\chi^{2}$ value | P value ^a^ (95.0%CI) |
| --- | --- | --- | --- | --- | --- | --- |
| Sex | | | | | 9.064 | .011 |
| Male | | 37(32.5%) | 58(50.9%) | 19(16.7%) |  |  |
| Female | | 102(48.8%) | 87(41.6%) | 20(9.6%) |  |  |
| Age(years) | | | | | 5.715 | .839 |
| 18–25 | | 3(42.9%) | 4(57.1%) | 0(0.0%) |  |  |
| 26–30 | | 19(41.3%) | 23(50.0%) | 4(8.7%) |  |  |
| 31–40 | | 50(43.9%) | 53(46.5%) | 11(9.6%) |  |  |
| 41–50 | | 47(42.7%) | 46(41.8%) | 17(15.5%) |  |  |
| 51–60 | | 19(43.2%) | 18(40.9%) | 7(15.9%) |  |  |
| More than 60 | | 1(50.0%) | 1(50.0%) | 0(0.0%) |  |  |
| Education | | | | | 7.673 | .263 |
| Doctor | | 38(42.2%) | 39(43.3%) | 13(14.4%) |  |  |
| Master's degree | | 52(40.9%) | 55(43.3%) | 20(15.7%) |  |  |
| Bachelor's degree | | 37(45.1%) | 41(50.0%) | 4(4.9%) |  |  |
| College degree and below | | 12(50.0%) | 10(41.7%) | 2(8.3%) |  |  |
| Hospital level | | | | | 13.238 | .039 |
| Tertiary A hospital | | 95(46.3%) | 89(43.4%) | 21(10.2%) |  |  |
| Tertiary hospital | | 16(27.6%) | 28(48.3%) | 14(24.1%) |  |  |
| Secondary hospital | | 18(45.0%) | 20(50.0%) | 2(5.0%) |  |  |
| First–level hospital and below | | 10(50.0%) | 8(40.0%) | 2(10.0%) |  |  |
| The Title of Dermatologists | | | | | 6.847 | .553 |
| Residents | | 30(45.5%) | 29(43.9%) | 7(10.6%) |  |  |
| Attending Physician | | 38(37.6%) | 52(51.5%) | 11(10.9%) |  |  |
| Deputy Chief Physician | | 39(45.3%) | 36(41.9%) | 11(12.8%) |  |  |
| Chief Physician | | 26(42.6%) | 25(41.0%) | 10(16.4%) |  |  |
| No title | | 6(66.7%) | 3(33.3%) | 0(0.0%) |  |  |

^a^ P values computed using the χ^2^ tests;

^b^ Data were analyzed on a total of 323 dermatologists, except the group of always use antioxidants (for the amount was too small to be statistically significant) (N=1).

Supplemental Table 2. Analysis of the Association Between the Demographic Variables and the Synergistic Curative Efficacy of Antioxidants.

| Characteristic ^b^ | Markedly effective | effective | uncertain | $\chi^{2}$ value | P value (95.0%CI) ^a^ |
| --- | --- | --- | --- | --- | --- |
| Sex | | | | 4.987 | .083 |
| Male | 11(14.5%) | 40(52.6%) | 25(32.9%) |  |  |
| Female | 8(7.5%) | 48(44.9%) | 51(47.7%) |  |  |
| Age(years) | | | | 10.538 | .395 |
| 18–25 | 0(0.0%) | 1(25.0%) | 3(75.0%) |  |  |
| 26–30 | 6(23.1%) | 10(38.5%) | 10(38.5%) |  |  |
| 31–40 | 4(6.3%) | 32(50.0%) | 28(43.8%) |  |  |
| 41–50 | 8(12.7%) | 31(49.2%) | 24(38.1%) |  |  |
| 51–60 | 1(4.0%) | 13(52.0%) | 11(44.0%) |  |  |
| More than 60 | 0(0.0%) | 1(100.0%) | 0(0.0%) |  |  |
| Education | | | | 2.346 | .885 |
| Doctor | 5(9.8%) | 24(47.1%) | 22(43.1%) |  |  |
| Master's degree | 8(10.7%) | 40(53.3%) | 27(36.0%) |  |  |
| Bachelor's degree | 5(11.1%) | 18(40.0%) | 22(48.9%) |  |  |
| College degree and below | 1(8.3%) | 6(50.0%) | 5(41.7%) |  |  |
| Hospital level | | | | 2.016 | .918 |
| Tertiary A hospital | 10(9.2%) | 50(45.9%) | 49(45.0%) |  |  |
| Tertiary hospital | 5(11.9%) | 23(54.8%) | 14(33.3%) |  |  |
| Secondary hospital | 3(13.6%) | 10(45.5%) | 9(40.9%) |  |  |
| First–level hospital and below | 1(10.0%) | 5(50.0%) | 4(40.0%) |  |  |
| The Title of Dermatologists | | | | 3.232 | .919 |
| Residents | 6(17.1%) | 15(42.9%) | 14(40.0%) |  |  |
| Attending Physician | 6(9.5%) | 30(47.6%) | 27(42.9%) |  |  |
| Deputy Chief Physician | 4(8.5%) | 24(51.1%) | 19(40.4%) |  |  |
| Chief Physician | 3(8.6%) | 18(51.4%) | 14(40.0%) |  |  |
| No title | 0(0.0%) | 1(33.3%) | 2(66.7%) |  |  |

^a^ P values computed using the χ^2^ tests;

^b^ Data were analyzed on 184 dermatologists who treated vitiligo with antioxidants, except the group of invalid effects (for the amount was too small to be statistically significant) (N=1).

Supplemental Table 3. Analysis of the Association Between the Knowledge of Antioxidants and the Frequency of the Use of Antioxidants.

| Characteristic ^b^ | Never | Occasionally | Frequently | $\chi^{2}$ value | P value (95.0%CI) ^a^ |
| --- | --- | --- | --- | --- | --- |
| Knowledge of antioxidants | | | | 15.020 | .001 |
| Insufficient | 76 (55.5%) | 48 (35.0%) | 13 (9.5%) |  |  |
| Sufficient | 63 (33.9%) | 97 (52.2%) | 26 (14.0%) |  |  |

^a^ P values computed using the χ^2^ tests;

^b^ Data were analyzed on a total of 323 dermatologists, except the group of always use antioxidants (for the amount was too small to be statistically significant) (N=1).

|  | Markedly effective | effective | uncertain | $\chi^{2}$ value | *P* value (95.0%CI) ^a^ |
| --- | --- | --- | --- | --- | --- |
| Consider using antioxidants in patients with stable progression | | | | 1.107 | .575 |
| disagree | 7(8.4%) | 43(51.8%) | 33(39.8%) |  |  |
| agree | 12(12.0%) | 45(45.0%) | 43(43.0%) |  |  |
| Consider using antioxidants in patients with advanced progression | | | | 11.986 | .002 |
| disagree | 6(5.8%) | 47(45.2%) | 51(49.0%) |  |  |
| agree | 14(12.8%) | 61(56.0%) | 34(31.2%) |  |  |
| Consider using antioxidants in patients with rapid progression | | | | 8.629 | .013 |
| disagree | 5(5.4%) | 27(43.5%) | 47(51.1%) |  |  |
| agree | 13(16.5%) | 41(51.9%) | 25(31.6%) |  |  |
| Consider using antioxidants in segmental vitiligo | | | | 8.184 | .017 |
| disagree | 4(5.5%) | 30(41.1%) | 39(53.4%) |  |  |
| agree | 15(13.6%) | 58(52.7%) | 37(33.6%) |  |  |
| Consider using antioxidants in non-segmental vitiligo | | | | 1.275 | .529 |
| disagree | 7(9.6%) | 32(43.8%) | 34(46.6%) |  |  |
| agree | 12(10.9%) | 56(50.9%) | 42(38.2%) |  |  |
| Consider using antioxidants in undetermined vitiligo | | | | 1.839 | .399 |
| disagree | 7(8.6%) | 36(44.4%) | 38(46.9%) |  |  |
| agree | 12(11.8%) | 52(51.0%) | 38(37.3%) |  |  |
| Consider using antioxidants in light vitiligo (area <1%) | | | | 1.415 | .493 |
| disagree | 10(9.2%) | 50(45.9%) | 49(45.0%) |  |  |
| agree | 9(12.2%) | 38(51.4%) | 27(36.5%) |  |  |
| Consider the use of antioxidants in moderate vitiligo (area 1%-5%) | | | | 11.747 | .003 |
| disagree | 5(7.9%) | 21(33.3%) | 37(58.7%) |  |  |
| agree | 14(11.7%) | 67(55.8%) | 39(32.5%) |  |  |
| Consider using antioxidants in moderate to severe vitiligo (area 6%-50%) | | | | 9.658 | .008 |
| disagree | 5(5.5%) | 39(42.9%) | 47(51.6%) |  |  |
| agree | 14(15.2%) | 49(53.3%) | 29(31.5%) |  |  |
| Consider using antioxidants in severe vitiligo (area> 50%) | | | | 5.156 | .076 |
| disagree | 8(7.3%) | 50(45.5%) | 52(47.3%) |  |  |
| agree | 11(15.1%) | 38(52.1%) | 24(32.9%) |  |  |

Supplemental Table 4. Analysis of the Association Between the Clinical Situations and the Synergistic Curative Efficacy of Antioxidants.

Continue to Supplemental Table 4

|  | Markedly effective | effective | uncertain | $\chi^{2}$ value | *P* value (95.0%CI) |
| --- | --- | --- | --- | --- | --- |
| Consider using antioxidants in 0–2 years old patients | | | | 7.636 | .022 |
| disagree | 15(8.9%) | 79(47.0%) | 74(44.0%) |  |  |
| agree | 4(26.7%) | 9(60.0%) | 2(13.3%) |  |  |
| Consider using antioxidants in 3–12 years old patients | | | | 2.801 | .246 |
| disagree | 14(9.4%) | 69(46.3%) | 66(44.3%) |  |  |
| agree | 5(14.7%) | 19(55.9%) | 10(29.4%) |  |  |
| Consider using antioxidants in 13–18 years old patients | | | | 7.844 | .020 |
| disagree | 10(8.8%) | 47(41.6%) | 56(49.6%) |  |  |
| agree | 9(12.9%) | 41(58.6%) | 20(28.6%) |  |  |
| Consider using antioxidants in ＞18 years old patients | | | | 1.829 | .401 |
| disagree | 4(19.0%) | 10(47.6%) | 7(33.3%) |  |  |
| agree | 15(9.3%) | 78(48.1%) | 69(42.6%) |  |  |
| Consider using antioxidants with topical therapy | | | | 2.978 | .226 |
| disagree | 10(7.9%) | 64(50.4%) | 53(41.7%) |  |  |
| agree | 9(16.1%) | 24(42.9%) | 23(41.1%) |  |  |
| Consider using antioxidants with oral therapy | | | | 4.884 | .087 |
| disagree | 13(15.9%) | 36(43.9%) | 33(40.2%) |  |  |
| agree | 6(5.9%) | 52(51.5%) | 43(42.6%) |  |  |
| Consider using antioxidants with oral and topical combination therapy | | | | 12.272 | .002 |
| disagree | 6(5.5%) | 48(44.0%） | 55(50.5%) |  |  |
| agree | 13(17.6%) | 40(54.1%） | 21(28.4%) |  |  |
| The course duration of antioxidants(months) | | | | 12.444 | .037 |
| <1 | 0(0.0%) | 3(18.8%） | 13(81.3%) |  |  |
| 1–3 | 12(9.8%) | 59(48.4%） | 51(41.8%) |  |  |
| 4–6 | 4(13.3%) | 16(53.3%） | 10(33.3%) |  |  |
| >6 | 3(20.0%) | 10(66.7%) | 2(13.3%) |  |  |

^a^ P values computed using the χ^2^ tests;

^b^ Data were analyzed on 184 dermatologists who treated vitiligo with antioxidants, except the group of invalid effects (for the amount was too small to be statistically significant) (N=1).

Supplemental Figure 2. Possible Clinical Situations Affect the Choice for the Use of Antioxidants.
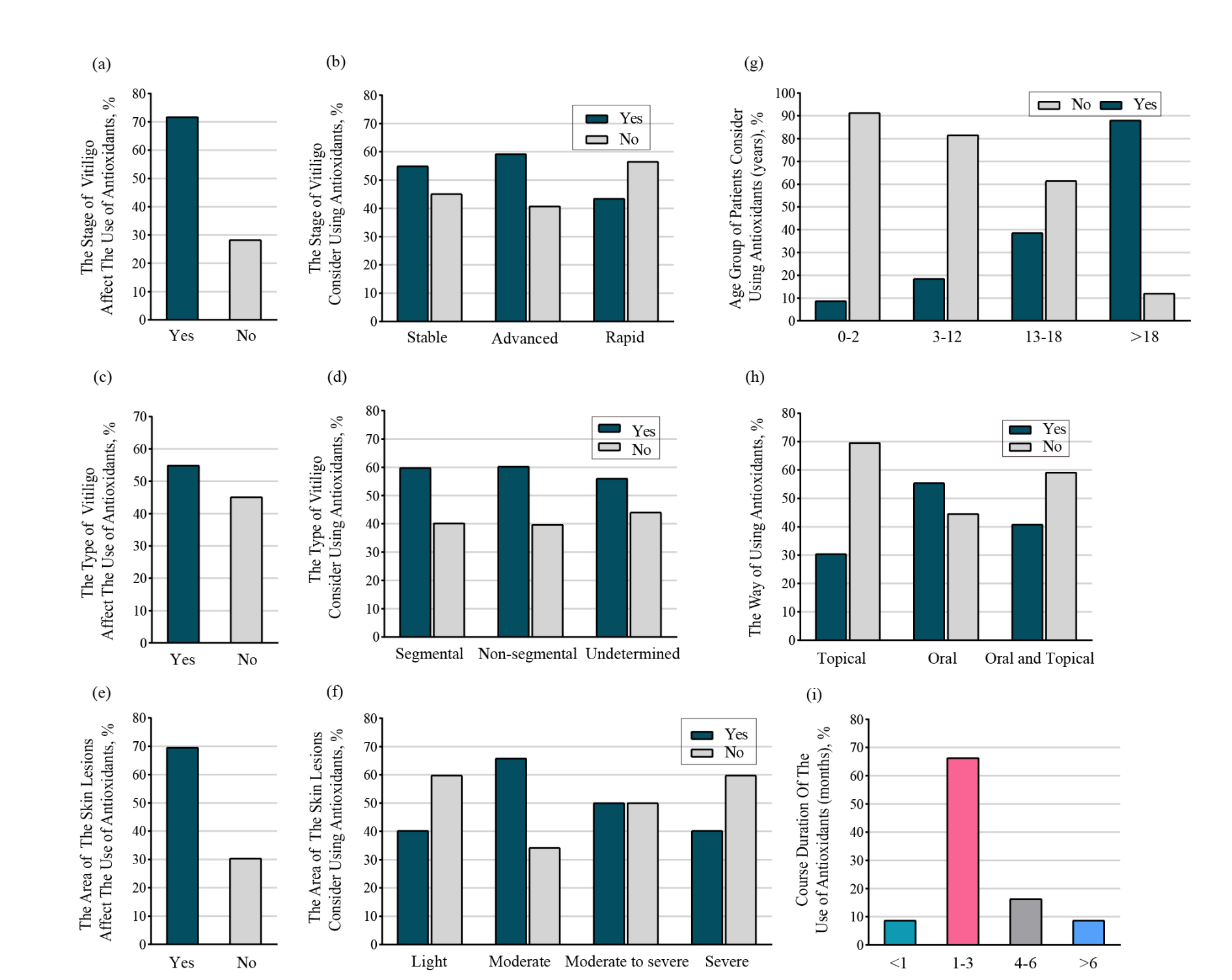


(a), The stage of vitiligo in a patient affect the choice for the use of antioxidants; (b), The stage of vitiligo consider using Antioxidants; (c), The type of vitiligo in a patient affect the choice for the use of antioxidants; (d), The type of vitiligo consider using Antioxidants; (e), The area of the skin lesions in a patient affect the choice for the use of antioxidants; (f), The area of the skin lesions consider using Antioxidants; (g), The age group of patients consider using antioxidants; (h), The use–pattern of antioxidants consider using antioxidants; (i), The course duration of the use of antioxidants.
