# Supplementary figures and images for: The Current Status of Antioxidants in the Treatment of Vitiligo in China
Source: Oxid Med Cell Longev. 2022 Feb 24;2022:2994558. doi: 10.1155/2022/2994558 (PMC8896159; doi:10.1155/2022/2994558)

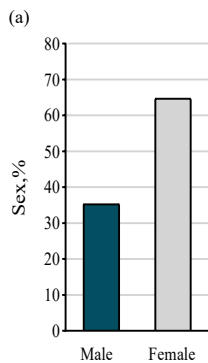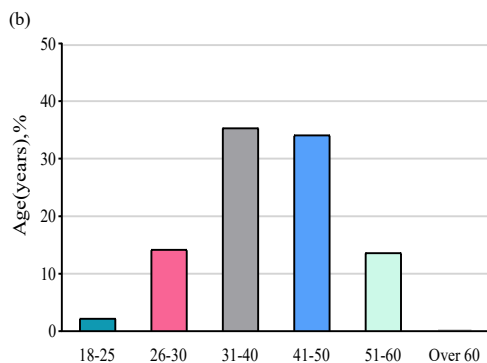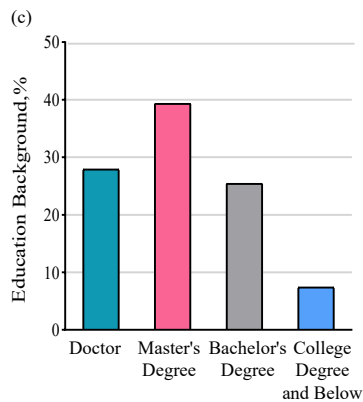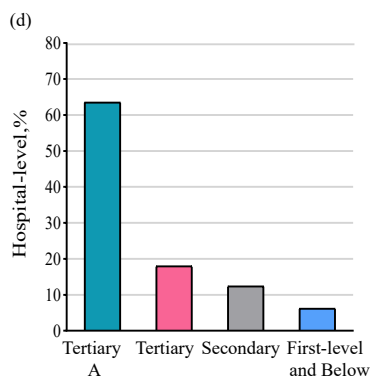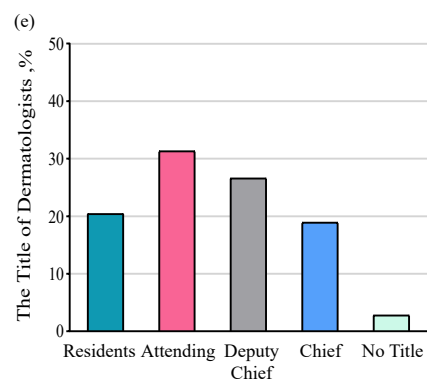

Supplement: Supplementary 2 — Supplemental Figure 1: the demographic distribution of the respondents. [file 2994558.f2.pdf]

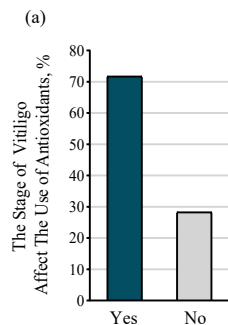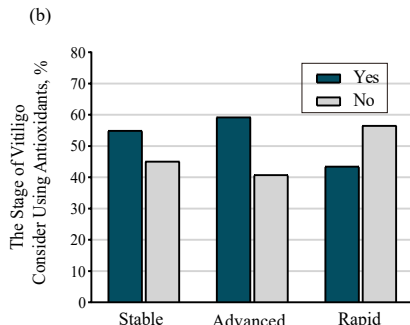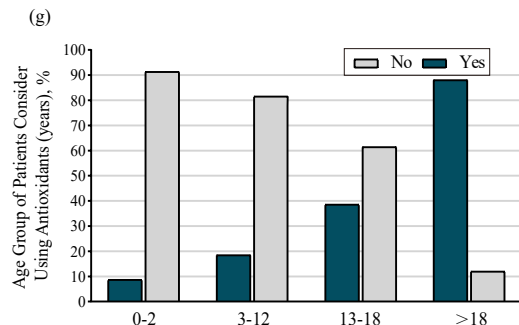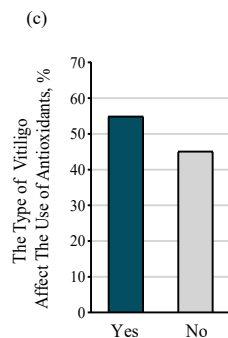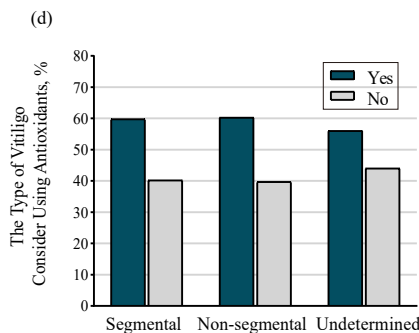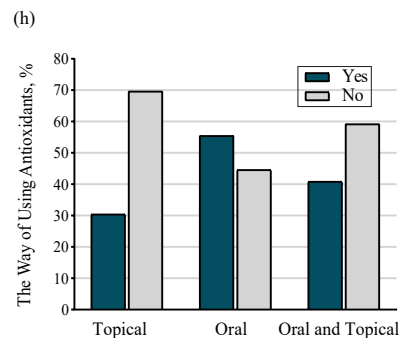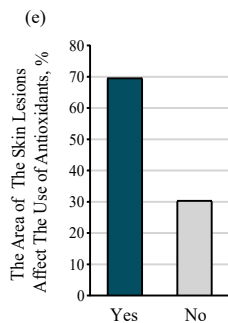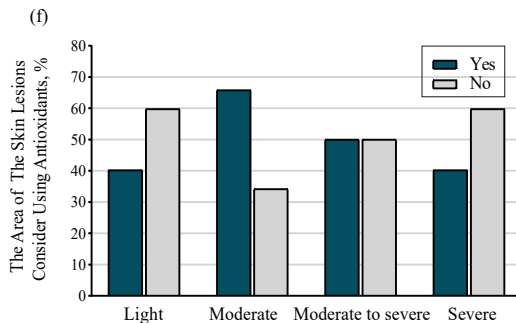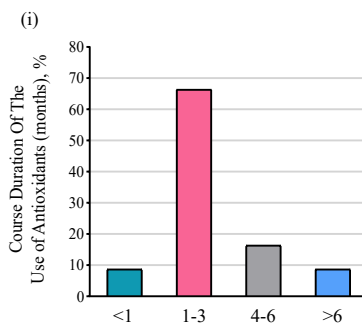

Supplement: Supplementary 7 — Supplemental Figure 2: possible clinical situations affect the choice for the use of antioxidants. [file 2994558.f7.pdf]
