# Supplementary material for: The Current Status of Antioxidants in the Treatment of Vitiligo in China
Source: Oxid Med Cell Longev. 2022 Feb 24;2022:2994558. doi: 10.1155/2022/2994558 (PMC8896159; doi:10.1155/2022/2994558)
Supplement: Supplementary 3 — Supplemental Table 1: analysis of the association between the demographic variables and the frequency of the use of antioxidants. [file 2994558.f3.docx]

Supplemental Table 1. Analysis of the Association Between the Demographic Variables and the Frequency of the Use of Antioxidants.

| Characteristic ^b^ | Never use antioxidants | | Use antioxidants occasionally | Always use antioxidants | $\chi^{2}$ value | P value ^a^ (95.0%CI) |
| --- | --- | --- | --- | --- | --- | --- |
| Sex | | | | | 9.064 | .011 |
| Male | | 37(32.5%) | 58(50.9%) | 19(16.7%) |  |  |
| Female | | 102(48.8%) | 87(41.6%) | 20(9.6%) |  |  |
| Age(years) | | | | | 5.715 | .839 |
| 18–25 | | 3(42.9%) | 4(57.1%) | 0(0.0%) |  |  |
| 26–30 | | 19(41.3%) | 23(50.0%) | 4(8.7%) |  |  |
| 31–40 | | 50(43.9%) | 53(46.5%) | 11(9.6%) |  |  |
| 41–50 | | 47(42.7%) | 46(41.8%) | 17(15.5%) |  |  |
| 51–60 | | 19(43.2%) | 18(40.9%) | 7(15.9%) |  |  |
| More than 60 | | 1(50.0%) | 1(50.0%) | 0(0.0%) |  |  |
| Education | | | | | 7.673 | .263 |
| Doctor | | 38(42.2%) | 39(43.3%) | 13(14.4%) |  |  |
| Master's degree | | 52(40.9%) | 55(43.3%) | 20(15.7%) |  |  |
| Bachelor's degree | | 37(45.1%) | 41(50.0%) | 4(4.9%) |  |  |
| College degree and below | | 12(50.0%) | 10(41.7%) | 2(8.3%) |  |  |
| Hospital level | | | | | 13.238 | .039 |
| Tertiary A hospital | | 95(46.3%) | 89(43.4%) | 21(10.2%) |  |  |
| Tertiary hospital | | 16(27.6%) | 28(48.3%) | 14(24.1%) |  |  |
| Secondary hospital | | 18(45.0%) | 20(50.0%) | 2(5.0%) |  |  |
| First–level hospital and below | | 10(50.0%) | 8(40.0%) | 2(10.0%) |  |  |
| The Title of Dermatologists | | | | | 6.847 | .553 |
| Residents | | 30(45.5%) | 29(43.9%) | 7(10.6%) |  |  |
| Attending Physician | | 38(37.6%) | 52(51.5%) | 11(10.9%) |  |  |
| Deputy Chief Physician | | 39(45.3%) | 36(41.9%) | 11(12.8%) |  |  |
| Chief Physician | | 26(42.6%) | 25(41.0%) | 10(16.4%) |  |  |
| No title | | 6(66.7%) | 3(33.3%) | 0(0.0%) |  |  |
