# Supplementary material for: The Current Status of Antioxidants in the Treatment of Vitiligo in China
Source: Oxid Med Cell Longev. 2022 Feb 24;2022:2994558. doi: 10.1155/2022/2994558 (PMC8896159; doi:10.1155/2022/2994558)
Supplement: Supplementary 4 — Supplemental Table 2: analysis of the association between the demographic variables and the synergistic curative efficacy of antioxidants. [file 2994558.f4.docx]

Supplemental Table 2. Analysis of the Association Between the Demographic Variables and the Synergistic Curative Efficacy of Antioxidants.

| Characteristic ^b^ | Markedly effective | effective | uncertain | $\chi^{2}$ value | P value (95.0%CI) ^a^ |
| --- | --- | --- | --- | --- | --- |
| Sex | | | | 4.987 | .083 |
| Male | 11(14.5%) | 40(52.6%) | 25(32.9%) |  |  |
| Female | 8(7.5%) | 48(44.9%) | 51(47.7%) |  |  |
| Age(years) | | | | 10.538 | .395 |
| 18–25 | 0(0.0%) | 1(25.0%) | 3(75.0%) |  |  |
| 26–30 | 6(23.1%) | 10(38.5%) | 10(38.5%) |  |  |
| 31–40 | 4(6.3%) | 32(50.0%) | 28(43.8%) |  |  |
| 41–50 | 8(12.7%) | 31(49.2%) | 24(38.1%) |  |  |
| 51–60 | 1(4.0%) | 13(52.0%) | 11(44.0%) |  |  |
| More than 60 | 0(0.0%) | 1(100.0%) | 0(0.0%) |  |  |
| Education | | | | 2.346 | .885 |
| Doctor | 5(9.8%) | 24(47.1%) | 22(43.1%) |  |  |
| Master's degree | 8(10.7%) | 40(53.3%) | 27(36.0%) |  |  |
| Bachelor's degree | 5(11.1%) | 18(40.0%) | 22(48.9%) |  |  |
| College degree and below | 1(8.3%) | 6(50.0%) | 5(41.7%) |  |  |
| Hospital level | | | | 2.016 | .918 |
| Tertiary A hospital | 10(9.2%) | 50(45.9%) | 49(45.0%) |  |  |
| Tertiary hospital | 5(11.9%) | 23(54.8%) | 14(33.3%) |  |  |
| Secondary hospital | 3(13.6%) | 10(45.5%) | 9(40.9%) |  |  |
| First–level hospital and below | 1(10.0%) | 5(50.0%) | 4(40.0%) |  |  |
| The Title of Dermatologists | | | | 3.232 | .919 |
| Residents | 6(17.1%) | 15(42.9%) | 14(40.0%) |  |  |
| Attending Physician | 6(9.5%) | 30(47.6%) | 27(42.9%) |  |  |
| Deputy Chief Physician | 4(8.5%) | 24(51.1%) | 19(40.4%) |  |  |
| Chief Physician | 3(8.6%) | 18(51.4%) | 14(40.0%) |  |  |
| No title | 0(0.0%) | 1(33.3%) | 2(66.7%) |  |  |
