# Supplementary material for: The Current Status of Antioxidants in the Treatment of Vitiligo in China
Source: Oxid Med Cell Longev. 2022 Feb 24;2022:2994558. doi: 10.1155/2022/2994558 (PMC8896159; doi:10.1155/2022/2994558)
Supplement: Supplementary 5 — Supplemental Table 3: analysis of the association between the knowledge of antioxidants and the frequency of the use of antioxidants. [file 2994558.f5.docx]

Supplemental Table 3. Analysis of the Association Between the Knowledge of Antioxidants and the Frequency of the Use of Antioxidants.

| Characteristic ^b^ | Never | Occasionally | Frequently | $\chi^{2}$ value | P value (95.0%CI) ^a^ |
| --- | --- | --- | --- | --- | --- |
| Knowledge of antioxidants | | | | 15.020 | .001 |
| Insufficient | 76 (55.5%) | 48 (35.0%) | 13 (9.5%) |  |  |
| Sufficient | 63 (33.9%) | 97 (52.2%) | 26 (14.0%) |  |  |
