# Supplementary material for: The Current Status of Antioxidants in the Treatment of Vitiligo in China
Source: Oxid Med Cell Longev. 2022 Feb 24;2022:2994558. doi: 10.1155/2022/2994558 (PMC8896159; doi:10.1155/2022/2994558)
Supplement: Supplementary 6 — Supplemental Table 4: analysis of the association between the clinical situations and the synergistic curative efficacy of antioxidants. [file 2994558.f6.docx]

Supplemental Table 4. Analysis of the Association Between the Clinical Situations and the Synergistic Curative Efficacy of Antioxidants.

|  | Markedly effective | effective | uncertain | $\chi^{2}$ value | *P* value (95.0%CI) ^a^ |
| --- | --- | --- | --- | --- | --- |
| Consider using antioxidants in patients with stable progression | | | | 1.107 | .575 |
| disagree | 7(8.4%) | 43(51.8%) | 33(39.8%) |  |  |
| agree | 12(12.0%) | 45(45.0%) | 43(43.0%) |  |  |
| Consider using antioxidants in patients with advanced progression | | | | 11.986 | .002 |
| disagree | 6(5.8%) | 47(45.2%) | 51(49.0%) |  |  |
| agree | 14(12.8%) | 61(56.0%) | 34(31.2%) |  |  |
| Consider using antioxidants in patients with rapid progression | | | | 8.629 | .013 |
| disagree | 5(5.4%) | 27(43.5%) | 47(51.1%) |  |  |
| agree | 13(16.5%) | 41(51.9%) | 25(31.6%) |  |  |
| Consider using antioxidants in segmental vitiligo | | | | 8.184 | .017 |
| disagree | 4(5.5%) | 30(41.1%) | 39(53.4%) |  |  |
| agree | 15(13.6%) | 58(52.7%) | 37(33.6%) |  |  |
| Consider using antioxidants in non-segmental vitiligo | | | | 1.275 | .529 |
| disagree | 7(9.6%) | 32(43.8%) | 34(46.6%) |  |  |
| agree | 12(10.9%) | 56(50.9%) | 42(38.2%) |  |  |
| Consider using antioxidants in undetermined vitiligo | | | | 1.839 | .399 |
| disagree | 7(8.6%) | 36(44.4%) | 38(46.9%) |  |  |
| agree | 12(11.8%) | 52(51.0%) | 38(37.3%) |  |  |
| Consider using antioxidants in light vitiligo (area <1%) | | | | 1.415 | .493 |
| disagree | 10(9.2%) | 50(45.9%) | 49(45.0%) |  |  |
| agree | 9(12.2%) | 38(51.4%) | 27(36.5%) |  |  |
| Consider the use of antioxidants in moderate vitiligo (area 1%-5%) | | | | 11.747 | .003 |
| disagree | 5(7.9%) | 21(33.3%) | 37(58.7%) |  |  |
| agree | 14(11.7%) | 67(55.8%) | 39(32.5%) |  |  |
| Consider using antioxidants in moderate to severe vitiligo (area 6%-50%) | | | | 9.658 | .008 |
| disagree | 5(5.5%) | 39(42.9%) | 47(51.6%) |  |  |
| agree | 14(15.2%) | 49(53.3%) | 29(31.5%) |  |  |
| Consider using antioxidants in severe vitiligo (area> 50%) | | | | 5.156 | .076 |
| disagree | 8(7.3%) | 50(45.5%) | 52(47.3%) |  |  |
| agree | 11(15.1%) | 38(52.1%) | 24(32.9%) |  |  |

Continue to Supplemental Table 4

|  | Markedly effective | effective | uncertain | $\chi^{2}$ value | *P* value (95.0%CI) |
| --- | --- | --- | --- | --- | --- |
| Consider using antioxidants in 0–2 years old patients | | | | 7.636 | .022 |
| disagree | 15(8.9%) | 79(47.0%) | 74(44.0%) |  |  |
| agree | 4(26.7%) | 9(60.0%) | 2(13.3%) |  |  |
| Consider using antioxidants in 3–12 years old patients | | | | 2.801 | .246 |
| disagree | 14(9.4%) | 69(46.3%) | 66(44.3%) |  |  |
| agree | 5(14.7%) | 19(55.9%) | 10(29.4%) |  |  |
| Consider using antioxidants in 13–18 years old patients | | | | 7.844 | .020 |
| disagree | 10(8.8%) | 47(41.6%) | 56(49.6%) |  |  |
| agree | 9(12.9%) | 41(58.6%) | 20(28.6%) |  |  |
| Consider using antioxidants in ＞18 years old patients | | | | 1.829 | .401 |
| disagree | 4(19.0%) | 10(47.6%) | 7(33.3%) |  |  |
| agree | 15(9.3%) | 78(48.1%) | 69(42.6%) |  |  |
| Consider using antioxidants with topical therapy | | | | 2.978 | .226 |
| disagree | 10(7.9%) | 64(50.4%) | 53(41.7%) |  |  |
| agree | 9(16.1%) | 24(42.9%) | 23(41.1%) |  |  |
| Consider using antioxidants with oral therapy | | | | 4.884 | .087 |
| disagree | 13(15.9%) | 36(43.9%) | 33(40.2%) |  |  |
| agree | 6(5.9%) | 52(51.5%) | 43(42.6%) |  |  |
| Consider using antioxidants with oral and topical combination therapy | | | | 12.272 | .002 |
| disagree | 6(5.5%) | 48(44.0%） | 55(50.5%) |  |  |
| agree | 13(17.6%) | 40(54.1%） | 21(28.4%) |  |  |
| The course duration of antioxidants(months) | | | | 12.444 | .037 |
| <1 | 0(0.0%) | 3(18.8%） | 13(81.3%) |  |  |
| 1–3 | 12(9.8%) | 59(48.4%） | 51(41.8%) |  |  |
| 4–6 | 4(13.3%) | 16(53.3%） | 10(33.3%) |  |  |
| >6 | 3(20.0%) | 10(66.7%) | 2(13.3%) |  |  |
